# Maternal body mass index and access to antenatal care: a retrospective analysis of 619,502 births in England

**DOI:** 10.1186/s12884-017-1475-5

**Published:** 2017-09-06

**Authors:** Charlotte Barber, Judith Rankin, Nicola Heslehurst

**Affiliations:** Institute of Health & Society, Newcastle University, Baddiley-Clark Building, Richardson Road, Newcastle upon Tyne, NE2 4AX UK

**Keywords:** Antenatal care, Late access, Inequalities, Body mass index, Obesity, Ethnic group, Teenage pregnancy, Deprivation, Employment

## Abstract

**Background:**

Late access to antenatal care increases risks of adverse outcomes including maternal and perinatal mortality. There is evidence that BMI influences patient engagement with health services, such as cancer screening services and delayed access to treatment; this association has not been fully explored in the context of antenatal care. This study investigated the association between the stage of pregnancy women access antenatal care, BMI, and other socio-demographic factors.

**Methods:**

Retrospective analysis of routine hospital data from 34 NHS maternity units in England, UK, including 619,502 singleton births between 1989 and 2007. Analyses used logistic regression to investigate the association between maternal BMI categories and stage of pregnancy women accessed antenatal care. Adjusted odds ratios (aORs) and 95% confidence intervals (CIs) were used to estimate associations, adjusting for maternal age, ethnic group, parity, Index of Multiple Deprivation score and employment status. The primary outcome was late access to antenatal care (>13^+6^ weeks). Secondary outcomes were trimester of access, and the association between late access and other socio-demographic variables.

**Results:**

Women with an overweight or obese BMI accessed antenatal care later than women with a recommended BMI (aOR 1.11, 95%CI 1.09–1.12; aOR 1.04, 95%CI 1.02–1.06 respectively), and underweight women accessed care earlier (aOR 0.77, 95%CI 0.74–0.81). Women with obesity were 42% more likely to access care in the third trimester compared with women with a recommended BMI. Additional significant socio-demographic associations with late access included women from minority ethnic groups, teenagers, unemployment and deprivation. The greatest association was observed among Black/Black British women accessing care in the third trimester (aOR 5.07, 95% CI 4.76, 5.40).

**Conclusions:**

There are significant and complex socio-demographic inequalities associated with the stage of pregnancy women access maternity care, particularly for women with obesity accessing care very late in their pregnancy, and among BME groups, teenagers, deprived and unemployed women. These populations are at increased risk of adverse maternal and fetal outcomes and require support to address inequalities in access to antenatal care. Interventions to facilitate earlier access to care should address the complex and inter-related nature of these inequalities to improve pregnancy outcomes among high-risk groups.

**Electronic supplementary material:**

The online version of this article (10.1186/s12884-017-1475-5) contains supplementary material, which is available to authorized users.

## Background

Late access to maternity services results in a lack of important antenatal care provision and intervention such as fetal anomaly screening, and is associated with adverse pregnancy outcomes including maternal and perinatal mortality and congenital anomalies [1–4]. National reports in the UK have identified that inadequate use of antenatal care was 15-fold higher among women who died during pregnancy compared with those who survived [5], and only one quarter of women who died between 2009 and 2014 received the recommended care according to national guidelines [6, 7]. This association with maternal mortality in the UK does not appear to have improved over the past 15 years as approximately one quarter of maternal deaths between 2003 and 2005 and 2006–2008 were among women who accessed antenatal care after 20 weeks gestation, or those who had limited or no antenatal care [8, 9]. These are potentially preventable maternal deaths.

Epidemiological analyses exploring associations between population characteristics and late access to antenatal care could inform targeted interventions and public health strategies to support high-risk populations. A systematic review published in 2003 explored inequalities in maternal attendance for antenatal care in the UK [10]. The authors described an absence of good quality published studies with a lack of consideration of confounders such as maternal age and parity in the analyses, and recommended further research to determine the extent of social inequalities in antenatal care [10]. Since this review was published, there have been multivariate analyses of localised UK populations published, including maternity populations in East London in the South of England (*n* = 20,135 births) and Sheffield in the North of England (*n* = 59,487 births); and univariate analyses of a postal survey of pregnant women accessing care in 15 maternity services in England (*n* = 839 women) [11–13]. These studies identified some socio-demographic inequalities in access to antenatal care among women from Black and Minority Ethnic (BME) groups, women born outside of the UK, young women aged under 20 years or teenage mothers, multiparity, not living with their partner, unemployment and deprivation [11–13].

There is a lack of research exploring the potential influence of maternal body mass index (BMI) on access to antenatal care. Maternal weight status is categorised, using early- or pre-pregnancy BMI, as underweight (<18.5 kg/m^2^), recommended weight (18.5–24.9 kg/m^2^), overweight (25–29.9 kg/m^2^) and obese (≥30 kg/m^2^). BMI is a significant predictor of maternal and perinatal health outcomes. For example, there is an increased risk of maternal and perinatal mortality, gestational diabetes, pre-eclampsia, pre- and post-term birth, stillbirth and congenital anomalies when mothers have a BMI in the overweight or obese ranges compared with women who have a BMI in the recommended range [7, 14–19]. There are also increased risks for mothers with an underweight BMI, including preterm birth and miscarriage [20–22]. There are similarities between some of the adverse outcomes associated with BMI, and those associated with late access to antenatal care. For example, 33% of mothers who died in 2012–2014 had an obese BMI and a further 18% an overweight BMI [7]. Epidemiological analyses of maternal obesity have also consistently shown significant associations with socio-demographic inequalities, similar to the inequalities in access to antenatal care such as deprivation and BME groups [23–25].

In addition to the similarity in socio-demographic factors and pregnancy outcomes among women with obesity and those accessing care late, there are also potential physiological and psycho-social associations. For example, there is a well-established association between obesity and delayed or avoidance of attendance for screening services, such as cervical and breast cancer screening [26, 27]. Pregnant women and non-pregnant patients describe delaying or avoiding accessing health services due to a history of negative interactions with health professionals about their weight, feeling blamed, and past experiences of obesity-related stigma within health service systems [27–31]. There is potential for this phenomenon of delayed access to health services among obese populations to extend into antenatal care, although this is an under explored area of research. From a physiological perspective, both underweight and obesity can contribute to irregular menstruation including oligomenorrhoea, amenorrhoea and irregular uterine bleeding [32, 33] which may contribute to delayed realisation and confirmation of pregnancy, and subsequent delayed access to care.

The potential associations between maternal weight status and delayed access to antenatal care warrants further exploration. This study aimed to establish the extent to which BMI is an independent factor in late access to antenatal care in a nationally representative sample of births in England, taking into consideration the influence of socio-demographic confounders.

## Methods

This study involved a secondary analysis of an existing nationally representative dataset of births in England, originally established to study trends in maternal BMI at booking (first antenatal contact) [24]. Methods of data collection for the national dataset are published elsewhere [23, 24]. The dataset included 738,307 singleton births between 1st January 1989 and 31st December 2007 in 34 maternity units in England, UK. For this study, data were excluded when the primary exposure variable (booking BMI) or outcome variable (gestational age at booking) were missing or unrealistic. Unrealistic BMI was defined as <13 kg/m^2^ or >80 kg/m^2^. Gestational age at booking was calculated using the date of booking, date of delivery, and gestational age at delivery. Unrealistic gestation at booking was defined as a negative gestational age (e.g. -10 weeks gestation at booking) or when it exceeded 44 weeks. The upper gestational age of 44 weeks was used as some women present for the first time during labour (unbooked pregnancies), and it is realistic that these pregnancies may have progressed up to 44 weeks if women were not engaged with antenatal care and therefore not offered any intervention such as induction for post-term birth, or women may have declined to be induced.

Adjustments to maternal booking BMI were applied to correct for naturally incurred gestational weight gain for women who booked after the 1st trimester (methods described in [24]). Pre-pregnancy BMI data are not routinely collected in the UK and the booking BMI is used as a proxy measure for pre-pregnancy weight status. BMI in the first trimester closely represents the pre-pregnancy weight status as maternal weight does not alter dramatically during this early stage of pregnancy. Therefore the BMI of women who booked in the first trimester was not adjusted. However, calculating BMI beyond the first trimester is less reflective of pre-pregnancy weight status due to the naturally incurred pregnancy-related weight gain, including the developing fetus, increased fluid volume and placenta as well as increased fat mass. BMI adjustments were required for women who booked beyond the first trimester to avoid false positives of maternal overweight and obesity which would inflate the association with late booking. The BMI change between the first trimester and the gestational week of booking for all women who booked after the first trimester was estimated using published data (described in [24]) and used to adjust the booking BMI to an estimate BMI which was more comparable to the women who booked in the first trimester. The maternal BMI exposure variable was categorised as underweight <18.5 kg/m^2^, recommended weight 18.5–24.9 kg/m^2^, overweight 25–29.9 kg/m^2^, and obese ≥30 kg/m^2^ [34]. Recommended BMI was the reference category for the analysis. The primary outcome variable was late access to antenatal care (late booking) defined as gestational age at booking >13^+6^ weeks, and compared with the reference category of ≤13^+6^ weeks [35]. The secondary outcome was trimester at booking defined as first trimester (weeks 1–13, reference category), second trimester (weeks 14–28) and third trimester (≥29 weeks) [36]. Additional socio-demographic variables were deprivation, ethnic group, employment, maternal age, and parity. Deprivation was defined using the Index of Multiple Deprivation (IMD) 2007 reference criteria. The IMD uses postcodes to calculate deprivation based on the area’s level of income deprivation, employment deprivation, health deprivation and disability, education, skills and training deprivation, barriers to housing and services, living environment deprivation, and crime. IMD scores were categorised into quintiles (where 1 = most deprived, and 5 = least deprived - reference category). Maternal ethnic groups were defined using national census categories of White (reference category), South Asian/Asian British, Black/Black British, Mixed Ethnic Group, and Chinese or Other Ethnic Group. Employment status was defined using UK census criteria: employed (reference category), not employed, housewife or carer, higher education, and school age/ in education under 18 years. Education variables were used in place of “not employed” rather than referring to educational attainment. Maternal age was defined as a dichotomous variable of teenage pregnancy less than 18 years of age, and not a teenage pregnancy (reference category). Parity categories were 0 (reference category), 1, 2, and 3 or more. These socio-demographic factors were included in the adjusted analyses as potential confounding variables for the association between maternal BMI and late access to care, and also included as secondary exposure variables to explore their independent association given the existing evidence-base from previous localised studies [11, 12].

Chi-squared tests were carried out to provide a descriptive summary of the dataset and the independent association between each exposure variable (BMI and socio-demographic factors) and the trimester of booking. Binary logistic regression analyses were used to examine associations between the exposure variable categories and late access to care. Model selection using a stepwise approach was carried out for the final adjusted logistic regression model. The same method of univariate and adjusted analyses were also carried out to explore associations between exposure variables and trimester of booking. A *p*-value of <0.05 was considered to be statistically significant and regression analyses are presented with 95% confidence intervals (CI). All data coding and statistical analyses were performed using SPSS version 21.

## Results

Following exclusions for missing or unrealistic data (16.1%) the dataset consisted of 619,502 births. In total, 89.8% of the 118,805 excluded cases were due to missing or invalid BMI data and 10.2% due to missing or invalid gestational age at booking. Analyses comparing the included and excluded populations suggested the data were missing at random (Additional file 1). Of the included population, 3.1% of women had an underweight BMI, 55.9% recommended BMI, 26.4% overweight, and 14.6% obese. The descriptive characteristics of the included population are shown in Table 1. All variables had a significant association with trimester at booking (*p* < 0.001). The proportion of women with a recommended or underweight BMI decreased as the booking trimester increased, whereas the proportion of women with an overweight or obese BMI increased with increasing booking trimester. A similar pattern of increasing proportion of women with each increase in booking trimester was observed for teenage pregnancies and for women aged 18–24 years; the BME groups Black or Black British, Chinese or Other Ethnic Group, and Mixed Ethnic Group; all categories of unemployment and women who were in education; and the second most deprived IMD Quintile 2 (Table 1).

**Table 1:** Socio-demographic characteristics of included population

	Total population (n)	First trimester booking (0–13 weeks) (n, %)	Second trimester booking (14–28 weeks) (n, %)	Third trimester booking (29–44 weeks) (n, %)	*p*
BMI Group					<0.001
Underweight	19,393	11,919 (3.4%)	6795 (2.9%)	679 (2.2%)	
Recommended	346,347	202,434 (56.9%)	129,353 (55.4%)	14,560 (47.7%)	
Overweight	163,282	90,431 (25.4%)	63,160 (27.1%)	9691 (31.8%)	
Obese	90,480	50,862 (14.3%)	34,053 (14.6%)	5565 (18.2%)	
Age Group (years)					<0.001
< 18	12,803	6230 (1.8%)	5657 (2.4%)	916 (3.0%)	
18–24	147,198	78,926 (22.2%)	59,986 (25.8%)	8286 (27.2%)	
25–30	207,699	120,695 (34.0%)	77,037 (33.1%)	9967 (32.8%)	
> 30	250,177	148,882 (42.0%)	90,047 (38.7%)	11,248 (37.0%)	
Teenage Pregnancy					<0.001
Yes	12,803	6230 (1.8%)	5657 (2.4%)	916 (3.0%)	
No	605,074	348,503 (98.2)	227,070 (97.6%)	29,501 (97.0%)	
Parity					<0.001
0	215,609	126,119 (36.4%)	77,817 (34.2%)	11,673 (40.2%)	
1	215,331	125,918 (36.4%)	79,939 (35.1%)	9474 (32.6%)	
2	102,061	57,908 (16.7%)	39,761 (17.5%)	4392 (15.1%)	
3+	69,552	36,076 (10.4%)	29,951 (13.2%)	3525 (12.1%)	
Ethnicity					<0.001
White	447,550	278,187 (88.2%)	149,341 (76.2%)	20,022 (74.5%)	
South Asian or Asian British	50,762	21,066 (6.7%)	26,957 (13.8%)	2739 (10,2%)	
Black or Black British	22,531	7571 (2.4%)	12,309 (6.3%)	2651 (9.9%)	
Chinese or Other Ethnic Group	11,399	5441 (1.7%)	4893 (2.5%)	1065 (4.0%)	
Mixed	5966	3174 (1.0%)	2394 (1.2%)	398 (1.5%)	
Employment					<0.001
Employed	262,556	167,707 (67.9%)	84,809 (57.8%)	10,040 (52.2%)	
Higher Education	8047	3845 (1.6%)	3670 (2.5%)	532 (2.8%)	
Housewife/ Carer	92,938	49,221 (19.9%)	38,463 (26.2%)	5254 (27.3%)	
Not employed	44,430	23,867 (9.7%)	17,595 (12.0%)	2968 (15.4%)	
School Age/ Education under 18 years	5092	2387 (1.0%)	2273 (1.5%)	432 (2.2%)	
IMD Quintile					<0.001
5- Least Deprived	125,474	79,022 (22.7%)	40,342 (18.5%)	6110 (20.8%)	
4	104,106	65,862 (18.9%)	33,490 (15.3%)	4754 (16.2%)	
3	110,058	65,790 (18.9%)	39,060 (17.9%)	5208 (17.8%)	
2	119,657	65,653 (18.9%)	47,590 (21.8%)	6414 (21.9%)	
1- Most Deprived	136,404	71,493 (20.6%)	58,058 (26.6%)	6853 (23.4%)	

All frequency (n) values exclude missing data, all % values use total valid responses (i.e. excluding missing values) as denominator

### Maternal booking BMI and late access to care

There were 36.5% (*n* = 226,234) of women who had a late booking (>13^+6^ weeks). Women with an underweight BMI were significantly less likely to book late than women with a recommended BMI, and women with an overweight or obese BMI were significantly more likely to book late (Table 2). All results remained significant following adjustment for socio-demographic variables, although the effect sizes reduced for all the BMI categories. Women with an overweight BMI were most likely to book late (aOR 1.11, 95% CI 1.09–1.12) (Table 2).

**Table 2:** Logistic regression analyses: association between maternal BMI, socio-demographic variables, and late booking (13^+6^ weeks)

	Unadjusted OR (95% CI)	Adjusted OR (95% CI)
BMI Group		
Recommended	Reference group	
Underweight	0.87 (0.85, 0.90)	0.77 (0.74, 0.81)
Overweight	1.15 (1.14, 1.17)	1.11 (1.09, 1.12)
Obese	1.13 (1.11, 1.14)	1.04 (1.02, 1.06)
Teenage pregnancy		
No	Reference group	
Yes	1.48 (1.43, 1.53)	1.43 (1.34, 1.53)
Parity		
0	Reference group	
1	1.00 (0.99, 1.02)	1.01 (0.99, 1.03)
2	1.09 (1.07, 1.10)	1.11 (1.08, 1.13)
3+	1.34 (1.32, 1.36)	1.28 (1.25, 1.31)
Ethnicity		
White	Reference group	
South Asian or Asian British	2.30 (2.26, 2.34)	2.32 (2.27, 2.39)
Black or Black British	3.11 (3.03, 3.20)	2.92 (2.82, 3.03)
Chinese or Other Ethnic Group	1.76 (1.70, 1.83)	1.76 (1.67, 1.85)
Mixed	1.45 (1.38, 1.53)	1.48 (1.39, 1.58)
Employment		
Employed	Reference group	
Higher Education	1.94 (1.86, 2.03)	1.39 (1.32, 1.46)
Housewife/ Carer	1.66 (1.63, 1.69)	1.31 (1.29, 1.33)
Not Employed	1.55 (1.52, 1.59)	1.31 (1.28, 1.34)
School Age/ Education under 18 years	2.13 (2.02, 2.25)	1.53 (1.40, 1.67)
IMD Quintile		
5-Least deprived	Reference group	
4	1.02 (1.00, 1.04)	0.99 (0.97, 1.02)
3	1.19 (1.17, 1.21)	1.05 (1.02, 1.07)
2	1.45 (1.43, 1.48)	1.12 (1.10, 1.15)
1- Most deprived	1.67 (1.64, 1.69)	1.29 (1.26, 1.32)

*Abbreviations*: *OR* odds ratio, *CI* confidence interval, *IMD* Index of Multiple Deprivation, *BMI* body mass index

In the analyses of the trimester that women booked for antenatal care, 57.4% (*n* = 355,646) of women booked in their first trimester, 37.7% (*n* = 233,361) in their second trimester, and 4.9% (*n* = 30,495) in their third trimester. Women booking in their second or third trimester were significantly less likely to have an underweight BMI and more likely to have an overweight or obese BMI (Table 3). The greatest effect size was observed among women with an overweight BMI in the analysis of booking in the second trimester (aOR 1.07, 95% CI 1.05, 1.08), and the association with obesity was not significant following adjustment for other socio-demographic variables (aOR 1.00, 95% CI 0.98–1.02). Larger effect sizes were observed for all BMI categories in the third trimester analyses than were observed for the second trimester analyses. The greatest effect size was observed among women with an obese BMI in the analysis of booking in the third trimester (aOR 1.42, 95% CI 1.36–1.48). All third trimester results remained significant following adjustment.

**Table 3:** Logistic regression analyses: association between maternal BMI, socio-demographic variables, and trimester at booking

	Second Trimester*		Third Trimester*	
	OR (95% CI)	aOR (95% CI)	OR (95% CI)	aOR (95% CI)
BMI Group				
Recommended	Reference group			
Underweight	0.89 (0.87, 0.92)	0.79 (0.76, 0.83)	0.79 (0.73, 0.86)	0.73 (0.66, 0.81)
Overweight	1.09 (1.08, 1.11)	1.07 (1.05, 1.08)	1.49 (1.45, 1.53)	1.39 (1.34, 1.44)
Obese	1.05 (1.03, 1.06)	1.00 (0.98, 1.02)	1.52 (1.47, 1.57)	1.42 (1.36, 1.48)
Teenage pregnancy				
No	Reference group			
Yes	1.39 (1.34, 1.45)	1.42 (1.33, 1.51)	1.74 (1.62, 1.86)	1.30 (1.13, 1.49)
Parity				
0	Reference group			
1	1.03 (1.02, 1.04)	1.06 (1.04, 1.08)	0.81 (0.79, 0.84)	0.75 (0.72, 0.78)
2	1.11 (1.10, 1.13)	1.17 (1.14, 1.19)	0.82 (0.79, 0.85)	0.70 (0.67, 0.74)
3+	1.35 (1.32, 1.37)	1.36 (1.32, 1.39)	1.06 (1.01, 1.10)	0.85 (0.80, 0.89)
Ethnicity				
White	Reference group			
South Asian or Asian British	2.38 (2.34, 2.43)	2.55 (2.49, 2.62)	1.81 (1.73, 1.88)	1.94 (1.83, 2.06)
Black or Black British	3.03 (2.94, 3.12)	2.92 (2.81, 3.04)	4.87 (4.64, 5.10)	5.07 (4.76, 5.40)
Chinese or Other Ethnic Group	1.68 (1.61, 1.74)	1.70 (1.62, 1.79)	2.72 (2.54, 2.91)	2.78 (2.54, 3.04)
Mixed	1.40 (1.33, 1.48)	1.45 (1.36, 1.54)	1.74 (1.57, 1.94)	1.81 (1.58, 2.06)
Employment				
Employed	Reference group			
Higher Education	1.89 (1.80, 1.98)	1.37 (1.30, 1.44)	2.31 (2.11, 2.54)	1.56 (1.40, 1.75)
Housewife/ Carer	1.55 (1.52, 1.57)	1.18 (1.15, 1.20)	1.78 (1.72, 1.85)	1.93 (1.85, 2.01)
Not Employed	1.46 (1.43, 1.49)	1.22 (1.20, 1.25)	2.08 (1.99, 2.17)	2.00 (1.91, 2.10)
School Age/ Education under 18 years	1.88 (1.78, 2.00)	1.36 (1.24, 1.49)	3.02 (2.72, 3.36)	2.58 (2.15, 3.10)
IMD Quintile				
5-Least deprived	Reference group			
4	1.00 (0.98, 1.01)	0.98 (0.96, 1.01)	0.93 (0.90, 0.97)	0.89 (0.85, 0.94)
3	1.16 (1.14, 1.18)	1.05 (1.02, 1.07)	1.02 (0.99, 1.06)	0.88 (0.83, 0.92)
2	1.42 (1.40, 1.44)	1.14 (1.12, 1.17)	1.26 (1.22, 1.31)	0.87 (0.82, 0.92)
1- Most deprived	1.59 (1.57, 1.62)	1.30 (1.28, 1.34)	1.24 (1.20, 1.29)	0.72 (0.69, 0.76)

* Trimester at booking compared with the reference group first trimester

*Abbreviations*: *OR* odds ratio, *CI* confidence interval, *aOR* adjusted odds ratio, *IMD* Index of Multiple Deprivation, *BMI* body mass index

### Additional socio-demographic factors and late access to care

There were significant associations with all of the additional socio-demographic variables included in the analyses for late booking, many of which had a greater effect size than the BMI categories. Following adjustments, women from BME groups, teenage pregnancies and women who were unemployed or in education were significantly more likely to book late (>13^+6^ weeks) (Table 2) or to book in the second or third trimester (Table 3). There was also a significant association with increasing parity and deprivation in the analysis of late booking (>13^+6^ weeks) and second trimester (Tables 2 and 3). However, in the third trimester analysis the observed direction of association for both parity and deprivation variables reversed following adjustments (Table 3). Black or Black British ethnic group had the greatest effect size for all analyses of late access, with similar results for late booking (aOR 2.92, 95% CI 2.82–3.03) and booking in the second trimester (aOR 2.92, 95% CI 2.81–3.04) compared with White women, and the greatest effect size for booking in the third trimester (aOR 5.07, 95% CI 4.76–5.40). Teenage pregnancies and mothers of school age or in education under the age of 18 years were also consistently associated with all categories of late access to care (Tables 2 and 3), with the greatest effect size observed among school age/education and third trimester booking (aOR 2.58, 95% CI 2.15–3.10). In addition to school age education, all other unemployed categories (i.e. not employed, housewife/carer, and higher education) were significantly associated with all late access outcomes following adjustments (Tables 2 and 3), and the effect size was greatest for unemployed women booking in the third trimester (aOR 2.00, 95% CI 1.91–2.10) (Table 3).

## Discussion

This study has identified significant associations between maternal BMI and the stage of pregnancy women book for antenatal care in England. Women with an overweight or obese BMI were more likely to access antenatal care later in pregnancy than women with an underweight or recommended BMI. A slightly increased association with booking beyond the first trimester was observed for women with an obese BMI, whereas the analysis of booking by trimester showed that the strongest association with maternal weight status and late booking was among women with obesity and booking very late in pregnancy, in the third trimester. Women who access antenatal care late are at increased risk of adverse outcomes, and this presents a double burden for women with obesity and their offspring who face risks due to both their weight status and due to late access. Accessing care late in pregnancy misses opportunities for routine screening such as the fetal anomaly scan at 20 weeks gestation [4]. As there is an established association between maternal obesity and congenital anomalies [18] this presents a missed opportunity for early detection for this population of women. Despite the significant associations between maternal BMI and late access to care identified in our study, these were not observed to the same extent as other socio-demographic factors including women from BME groups, teenagers and unemployed mothers. Other studies in England have reported similar associations with late access among BME groups, young women and unemployment [11–13]. Similar to obesity, both unemployment and BME groups are overrepresented among maternal deaths in the UK, [6, 9], identifying further inequalities in maternal and perinatal risk for these populations. In our study, 36.5% of women accessed antenatal care late which is comparable to local datasets for East London and Sheffield (37.5 to 49.9%) when taking into consideration national variation in BMI and other socio-demographic factors [11, 12]. However, there is limited comparative robust research specifically investigating maternal BMI and late access. A study in the USA found that women with an obese BMI accessed care 0.2 weeks later than women with a recommended BMI, although this was not statistically significant [37]. Similar to our findings, the authors identified that late or no access to antenatal care was more likely among women from BME groups, teenagers and multiparous mothers [37]. A further London-based study also identified that teenagers and multiparous women accessed care beyond 18 weeks, but the authors reported a high level of missing BMI data [38].

A recent review of the pathophysiology of obesity and menstrual disorders identified that obesity, especially central adiposity, was associated with increased oestrogen levels, circulating free testosterone, and with insulin levels which stimulates the production of androgens in ovarian tissue which can cause disruptions to normal ovulation and menstrual bleeding [32]. Additionally, the association with menstruation disturbances was stronger for early onset obesity potentially due to the leptin levels which regulates the gonadotropin surge initiating pubertal stages [32]. The association with menstrual disturbances may further contribute to late access to antenatal care due to delayed realisation about conception, particularly among women with central adiposity, or those who developed obesity during childhood. Qualitative research has also identified complex reasons for late access among socially excluded “hard to reach” groups of women. Haddrill et al. [39] reported three themes to describe reasons for delayed access to antenatal care, including *“not knowing”* where women reported a lack of realisation about the pregnancy or beliefs that they were not pregnant (e.g. due to contraception use or maternal age); *“knowing”* including women who avoided or postponed access to antenatal care (e.g. due to fears or lack of perceived value of antenatal care); and being *“delayed”* including health professional and healthcare system failures (e.g. mis-estimation of gestational age, delays with referrals and appointments). A study for the UK Department of Health also grouped women into two distinct typologies: those who embraced their pregnancy such as women from Asian, Muslim, Somali and Romany communities; and those who were anxious about their pregnancy such as women who were homeless, drug and alcohol dependent, with learning difficulties and teenagers [40]. The authors reported different reasons for delaying access to antenatal care between groups. Among those who embraced their pregnancy the family was a significant factor in their lives, and there were culturally defined roles of motherhood within society. Women were expected to continue their daily routine rather than seeking medical help as pregnancy was considered to just be part of life, or an act of God which was out of their hands. There was an emphasis on seeking medical advice from the family and distrust among certain groups of medical professionals [40]. Our study identified the strongest associations with late access among women from BME groups, and previous studies in the UK report that South Asian women had fewer antenatal appointments and waited longer before seeking antenatal care when compared with White women [10, 41], and women from BME groups report being insufficiently involved in decisions about their maternity care to have confidence and trust in the staff [42]. However, among the population categorised as being anxious, reasons for late access related to difficulties accepting the pregnancy, or that accessing care was less important than other priorities such as finding housing [40]. Similar to the group of women who embraced pregnancy, there was some distrust of medical professionals, plus fears of being labelled or referred to social services and having their baby taken into care. Extremely anxious women waited longer to seek antenatal care, particularly teenager mothers who reported feeling concerned about the associated stigma, fearful of health professionals informing their parents of the pregnancy, and relinquishing control [39, 40, 43]. Previous studies have reported that teenage mothers are less likely to keep appointments or attend antenatal classes, and access maternity care later [44, 45], which is also reflected in our analyses of teenage mothers and late access to care. Additional barriers to accessing care are inability to travel to appointments, language barriers for women who do not speak English, women with no fixed address who are not registered with a GP, and women with learning difficulties report being embarrassed to seek help as they did not understand written information leaflets [40, 43]. Although we were able to explore teenage pregnancy and maternal ethnic group in our analyses, the dataset did not include variables reflecting additional factors such as ability to speak English, learning difficulties or homelessness and therefore we could not explore these factors.

Delivery of maternity services and targeted public health support should encourage early access to antenatal care for optimal pregnancy outcomes for both mothers and babies. The findings of this research, and the existing qualitative research, highlight the complexity of socio-demographic inequalities associated with late access to antenatal care which are often interrelated. For example, women who are unemployed are more likely to have a higher BMI, as are women from more deprived areas and among BME groups [23, 25]. It is, therefore, important to try to tackle these inequalities in access to antenatal care in a way which holistically targets disadvantaged populations. Future epidemiological research investigating predictors of late access to antenatal care, and maternity or public health interventions to improve access to care, should consider the relationships between these complex factors particularly maternal BMI, employment, teenage pregnancy and ethnic minority groups.

### Strengths and limitations

This is the first national-level study to explore maternal BMI and other socio-demographic factors which may be associated with late access to antenatal care in the UK, considering the influence of confounders. The research has a large sample size of over 600,000 births, which is comparable to the national average pregnancy population in terms of maternal characteristics such as age [24]. The dataset used in this study is the only existing national, general maternity dataset (i.e. not restricted to mortality or other sub-population data) which incorporates maternal BMI data over a prolonged time period. A limitation of this study is the lack of data available post-2007. When comparing maternal BMI distribution in this dataset with recent UK cross sectional booking data, maternal overweight and obesity prevalence appear to have increased (obesity reported as 21% of all women booking in July in 2015, [46]). Therefore, the prevalence of late access relating to overweight and obesity is likely to be higher today than that reported in this study. A further limitation of this study relates to the nature of secondary analysis of a dataset which was collected for a different purpose. While existing datasets provide relatively instant access to a wealth of data for research which would take decades to prospectively collect, they are often limited as they lack the entire group of variables that would be required to fully answer the research question. For example, in this study it was not possible to explore the impact of some potentially important confounders that have been identified in the existing literature, such as women’s ability to speak English or the influence of a learning disability. Additionally, reliance on routine maternity data means it is not possible to identify whether the BMI data represents self-reported pre-pregnancy weight or measured booking pregnancy weight. UK guidelines state that the booking BMI should be measured [47]. However, a national report highlighted that 16% of maternity units used self-reported height and weight measurements rather than measured [25]. Self-reported weight is often underestimated and this may have caused an underrepresentation of overweight and obesity in the sample. Alternatively, if measured weights were used to define booking BMI then this would over-estimate overweight and obesity prevalence among women accessing care late in pregnancy as the measurements would also incorporate the naturally incurred weight gain of pregnancy (including fat gain, as well as the healthy weight of the fetus, placenta and fluids). Attempts to limit the effect of false positives of overweight and obesity were made by adjusting the BMI data for women who booked after the first trimester of pregnancy.

## Conclusions

Women with an obese or overweight BMI at booking were more likely to access antenatal care later in pregnancy than women who are of recommended weight or underweight, and are therefore more likely to be at increased risk of associated adverse maternal and fetal outcomes. Additional socio-demographic inequalities were observed among BME groups, teenagers and unemployed women. Targeted support is required to address inequalities in access to antenatal care to encourage earlier access and optimum outcomes among high-risk groups. Future research, public health and maternity interventions should aim to identify ways to engage with women from these high risk groups earlier in pregnancy, considering the complex and inter-related nature of the associated inequalities.

## Additional file

Additional file 1: Comparison of included and excluded data. A comparison of the characteristics of the included and excluded population, including the exposure variables (maternal BMI, other sociodemographic variables) and outcome variables (late booking and trimester of booking) (XLSX 14 kb)

## Abbreviations

aOR: Adjusted odds ratio
BME: Black and Minority Ethnic groups
BMI: Body mass index
CMACE: Centre for Maternal and Child Enquiries
CMACH: Centre for Maternal and Child Health
DH: Department of Health
IMD: Index of Multiple Deprivation
MBRRACE: Mothers and Babies: Reducing Risk through Audits and Confidential Enquiries across the UK
NICE: National Institute for Health and Care Excellence
OR: Odds ratio
